# The Full Breadth of Mendel’s Genetics

**DOI:** 10.1534/genetics.116.196626

**Published:** 2016-12-06

**Authors:** Peter J. van Dijk, T. H. Noel Ellis

**Affiliations:** * Keygene N.V., 6708 PW Wageningen, The Netherlands

**Keywords:** Gregor Mendel, genetics, *Hieracium*, constant hybrids, apomixis

## Abstract

Gregor Mendel’s “Experiments on Plant Hybrids” (1865/1866), published 150 years ago, is without doubt one of the most brilliant works in biology. Curiously, Mendel’s later studies on *Hieracium* (hawkweed) are usually seen as a frustrating failure, because it is assumed that they were intended to confirm the segregation ratios he found in *Pisum*. Had this been his intention, such a confirmation would have failed, since, unknown to Mendel, *Hieracium* species mostly reproduce by means of clonal seeds (apomixis). Here we show that this assumption arises from a misunderstanding that could be explained by a missing page in Mendel’s first letter to Carl Nägeli. Mendel’s writings clearly indicate his interest in “constant hybrids,” hybrids which do not segregate, and which were “essentially different” from “variable hybrids” such as in *Pisum*. After the *Pisum* studies, Mendel worked mainly on *Hieracium* for 7 years where he found constant hybrids and some great surprises. He also continued to explore variable hybrids; both variable and constant hybrids were of interest to Mendel with respect to inheritance and to species evolution. Mendel considered that their similarities and differences might provide deep insights and that their differing behaviors were “individual manifestations of a higher more fundamental law.”

The publication of Mendel’s letters to Carl Nägeli by Correns in 1905 was a service to genetics which seems not to have been fully appreciated by most of those who have since written accounts of Mendel’s life and work (Mann Lesley 1927).

“THESE [seedlings] have rooted well, and should flower next year. Whether they will *retain the characteristics of the hybrid*, *or whether they will show variations*, will be determined by next year’s observations” (our emphasis). These lines about the progeny of his first artificial hawkweed (*Hieracium*) hybrid were written by Gregor Mendel on November 6, 1867, in a letter to Carl Nägeli, professor of botany at Munich (Letter III, Stern and Sherwood 1966, p. 73). They indicate that from the beginning of his experiments with *Hieracium*, Mendel expected that constant-hybrid offspring may well occur. Mendel ends the letter with: “I look forward to the coming summer with impatience since the progeny of several fertile hybrids will bloom for the first time. They should be very numerous and I only hope that they repay the yearning [*Sehnsucht*!] with which I await them with much information concerning their life histories.” (quoted in Mann Lesley 1927). These are not the words of a frustrated man.

Gregor Mendel’s fame is based on his *Pisum* (pea) crossing experiments that were published 150 years ago. His only subsequent publication on plants is a preliminary communication on artificial *Hieracium* hybrids (Mendel 1870). The usual supposition about Mendel’s *Hieracium* experiments, which were carried out over 7 years, is that they were intended to verify the results he obtained with his *Pisum* experiments (Nogler 2006; Bicknell *et al.* 2016). Hawkweeds are related to dandelions and, like them, often reproduce by a peculiar and rare breeding system called apomixis. The seeds of apomictic plants are produced clonally and are thus genetically identical to the mother plant. This is achieved by the avoidance of meiosis and the parthenogenetic development of the egg cell. In apomictic hawkweeds, most seeds produced are apomictic, but some may develop after cross-fertilization (for more information on apomixis see Supplemental Material, Section 1, File S1). Hawkweeds are hermaphrodites and produce haploid pollen, so they can act as pollen donors in crosses. Thus the prevalence of apomixis in *Hieracium* would have made it impossible for Mendel to replicate his *Pisum* findings in this genus. Apomixis was unknown in Mendel’s time; indeed it was many years after his death that the Danish botanist Carl Hansen Ostenfeld (1904) discovered apomixis in *Hieracium*. The usual interpretation of Mendel’s *Hieracium* experiments then is that his work on this genus was a frustrating failure; we suggest this misinterprets Mendel’s purpose.

In “Experiments on Plant Hybrids” Mendel (1866) gives an exemplary description of the formation of hybrids and the diversity among their offspring. Most of the work concerns *Pisum*, but he confirmed his findings in the genus *Phaseolus* (common bean). When self-fertilized, F_1_ hybrids within these species produce variable progeny. Toward the end of this article, Mendel contrasts his results with the case where “We encounter an *essential difference* in those hybrids that remain constant in their progeny and propagate like pure strains.” (Mendel 1866; Stern and Sherwood 1966, p. 41. Mendel used “*reinen Arten*”, so “pure species” would be a better translation than “pure strains”). When self-fertilized, F_1_ hybrids of these other species breed true: their progeny do not vary. Mendel designated these two distinct classes as variable hybrids (Stern and Sherwood 1966, p. 42) and constant hybrids (Stern and Sherwood 1966, p. 41), respectively^1^.

Historians of science (*e.g.*, Olby 1979, 1985, 1997; Callender 1988; Müller-Wille and Orel 2007) have argued that Mendel’s main motivation for the *Hieracium* (and *Pisum*) experiments was his interest in hybridization and speciation rather than the inheritance of traits, and they proposed that Mendel stands in the tradition of earlier plant hybridizers like Joseph Gottlieb Kölreuter (1733–1806) and Carl Friedrich Gärtner (1772–1850). Recently this “Mendel as a nongeneticist” view has received considerable attention in popular science books (*e.g.*, Endersby 2007; Numbers and Kampourakis 2015) and education journals (*e.g.*, Peterson and Kampourakis 2015). Although we agree with these historians of science that Mendel selected *Hieracium* to study constant hybrids, we do not think that speciation by hybridization was his only or main motivation. Mendel was also interested in reproductive cells and segregation *vs.* nonsegregation in the successive generations of progeny from a hybrid (*i.e.*, inheritance). Mendel had multiple reasons for selecting *Hieracium* as an object for experimental crossing and the importance of these reasons may have shifted over the years of his study. The opportunity to come into contact with Carl Nägeli, the person most likely to value his *Pisum* findings, would have been additionally attractive.

In addition to his articles, there is a series of 10 letters that record part of his communication with Nägeli. Mendel’s notebooks were destroyed after his death, so we must rely on these few documents to form an understanding of his scientific thoughts and motives. From these documents we know that after *Pisum* and *Phaseolus*, Mendel investigated many other species from the genera *Aquilegia*, *Antirrhinum*, *Calceolaria*, *Campanula*, *Cheiranthus*, *Cirsium*, *Dianthus*, *Geum*, *Hieracium*, *Ipomoea*, *Linaria*, *Lychnis*, *Matthiola*, *Mirabilis*, *Tropaeoleum*, *Verbascum*, *Zea*, and more were planned (Letter II). By far, the largest number of these experiments was conducted in *Hieracium* (Cetl 1971). In this article, we argue that a (mis)reading of Mendel’s first letter to Nägeli has led to the incorrect idea that Mendel’s *Hieracium* experiments were intended to verify his *Pisum* findings.

## Correspondence Between Mendel and Nägeli

### Carl Nägeli

Carl Nägeli was one of the most important botanists of the 19th century (Junker 2011). His research interests were on natural hybrids, an area where he was recognized as the leading researcher; and *Hieracium*, where again he was the leading authority. Nägeli was the person who could best see the relevance of Mendel’s pea results and Mendel also wanted his advice as a *Hieracium* expert (Section 2, File S1).

### Mendel’s letters to Nägeli

Carl Correns (1900), one of the three “rediscoverers” of Mendel’s work, clearly acknowledged Mendel’s contribution. Correns was a student of Nägeli’s and (after Nägeli’s death) was married to his niece. From Mendel’s *Hieracium* note and from conversations with Nägeli in the past, Correns knew that Mendel and Nägeli had collaborated closely, so he asked the Nägeli family whether they had any letters from Mendel. Correns published the 10 letters that were discovered (Correns 1905), labeling them with the Roman numerals I to X (Table S1). In 1925, Correns wrote in a letter to Herbert Fuller Roberts that these “first came to light through an accident in 1904” (Roberts 1929, p. 338). Fragments of some of Nägeli’s letters to Mendel were found in the monastery in Brno (German: Brünn) and were published by Iltis (1924). The records of their correspondence are thus incomplete. Correns also published some of the keyword summaries that Nägeli had made of his letters to Mendel. The only in-depth analysis of this scientific correspondence we are aware of is Hoppe (1971), in which she discusses it especially in relation to Nägeli’s work, but not in relation to Mendel’s *Hieracium* results.

### Mendel’s *Hieracium* work has been misunderstood as a frustrating failure to replicate his *Pisum* work

The traditional interpretation of Mendel’s motivation for studying *Hieracium* is expressed by Hartl and Orel (1992): Mendel’s “studies of *Hieracium* and other species were undertaken to verify, with other plants, the result obtained with *Pisum*,” and “the experiments with *Hieracium*, as recounted in the letters to Nägeli, were one long chronicle of failure and frustration.” In 2006 the journal *GENETICS* marked the 140-year jubilee of Mendel’s *Pisum* article. Crow and Dove (in Nogler 2006) commented negatively about Mendel’s *Hieracium* work: “Here, on this anniversary, instead of extolling his success, we present a scholarly account [Nogler 2006] of Mendel’s frustrating attempts to repeat his findings in another species, which, unbeknownst to him, reproduced apomictically.” Nogler (2006) starts with: “Mendel hoped that the highly polymorphic genus *Hieracium* would be particularly promising for verifying the laws of inheritance that he had discovered while working on *Pisum*.” According to Mawer (2006, p. 167), Mendel’s *Hieracium* article is “of no more than curiosity value.” Modern articles on the genetics of apomixis often refer to Mendel’s frustrating experiences with *Hieracium* *e.g.*, Koltunow *et al.* (2011): “Apomixis in hawkweed: Mendel’s experimental nemesis.” At the Mendel Museum at the Monastery in Brno, Mendel’s *Pisum* experiments, meteorological studies, and beekeeping activities can be seen, but not his *Hieracium* work, perhaps due to their associated negativity.

It has been argued that Nägeli was instrumental in Mendel’s selection of *Hieracium* (as discussed in Nogler 2006), but from Letter I it is clear that Mendel had already made crosses in *Hieracium*, *Geum*, and *Cirsium* in the summer of 1866, so the parental species must have been collected at least one season earlier. Mendel had thus embarked on his *Hieracium* experiments by 1865 at the latest. Therefore Nägeli cannot have pushed Mendel to work on *Hieracium* as is sometimes suggested (Iltis 1924; Mayr 1982); his choice of *Hieracium* predates his communication with Nägeli and Nägeli’s expertise with *Hieracium* was a likely motivation for Mendel initiating this correspondence.

### Contradiction in Mendel’s first letter to Nägeli

Mendel’s first letter to Nägeli, written on New Year’s Eve 1866, was a covering letter for the reprint of his *Pisum* article. In the letter (Letter I) Mendel clarified his *Pisum* studies, mentioned his future research plans, and asked if he could rely on Nägeli for the determination of difficult *Hieracium* and *Cirsium* (thistle) species, on which Nägeli was an expert. To understand why it is widely believed that Mendel chose *Hieracium* to test the *Pisum* findings, paragraphs four and five are crucial, so these are copied below with the paragraph numbers added in parentheses:

(4) In order to determine the agreement, if any, with *Pisum*, a study of those forms which occur in the first generation^2^ should be sufficient. If, for two differentiating characters, the same ratios and developmental series which exist in *Pisum* can be found, the whole matter would be decided. Isolation during the flowering period should not present many difficulties in most cases, since we are dealing only with few plants; those plants whose flowers are being fertilized and a few hybrids which have been selected for seed production. Those hybrids which are collected in the wild can be used as secondary evidence only, as long as their origin is not unequivocally known.

(5) *Hieracium*, *Cirsium*, and *Geum* I have selected for further experiments. In the first two, manipulation in artificial pollination is very difficult and unreliable because of the small size and peculiar structure of the flowers . . . (Stern and Sherwood 1966, p. 57–58).

From this it has been concluded that Mendel chose the genera *Hieracium*, *Cirsium*, and *Geum* to test the *Pisum* findings. William Bateson (1909, p. 246) wrote: “This genus [*Hieracium*] being one of the most strikingly polymorphic, he chose it after his discovery regarding the inheritance of peas, as the subject of *further* [our emphasis] research. We may surmise that he expected to find in it illustrations of the new principles.” Bateson’s use of the word “further” suggests that he came to this conclusion based on the two paragraphs mentioned above^3^. This interpretation has become the common belief of geneticists. For example, Iltis (1924, translation of Iltis 1966) wrote: “For Mendel the behavior of the hawkweeds remained an enigma, and his experiments upon these composites shattered the hopes he had entertained of finding confirmation of the principles of inheritance worked out by him in the case of *Pisum*, and thus establishing these principles as universally valid general laws. . . . He had certainly been lucky in his original choice of *Pisum* as the object of his experiments. But fate played him an ill turn when he went on to hybridize the hawkweeds; and when, with peasant doggedness, urged on by Nägeli, he persevered so long in his researches upon this unsuitable genus.” (pp. 174–175). Ernst Mayr (1982, p. 723) stated: “Instead, he [Nägeli] encouraged Mendel to test his theory of inheritance in the hawkweeds (*Hieracium*), a genus in which, as we now know, parthenogenesis [apomixis] is common, leading to results that are incompatible with Mendel’s theory. In short, as one historian has put it, ‘Mendel’s connection with Nägeli was totally disastrous.’ ”

Was it ill fate, as Iltis suggested? One of the very few who has interpreted this differently is the historian L.A. Callender (1988), who wrote: “Mendel, on the other hand, and before he was certain that he had obtained a single *Hieracium* hybrid surmised exactly the opposite [of Bateson’s proposal that Mendel expected to verify his *Pisum* results]” and cites a later paragraph from Letter I: “The plant *Geum urbanum* + *rivale* deserves special attention. This plant, according to Gärtner (1849), belongs to the few so far^4^ known hybrids which produce nonvariable progeny as long as they remain self-pollinated.” And subsequently: “The surmise that some species of *Hieracium*, if hybridized, would behave in a fashion similar to *Geum*, is perhaps not without foundation. It is, for instance, very striking that the bifurcation of the stem, which must be considered an intermediate^5^ trait among the *Piloselloids*, may appear as a perfectly constant character, as I was able to observe last summer on seedlings of *H. stoloniflorum* W. K.^6^”

This suggests that Mendel expected that *Hieracium* species could be constant hybrids (see also Orel 1998). Why would Mendel select a genus in which he expected to find constant hybrids, to validate the segregation of variable hybrids? This would be irrational. The eminent Mendel-expert Franz Weiling (1970) expressed it very carefully: “From Mendel’s first letter to Nägeli one gets the impression that he, with his crosses in *Hieracium*, *Cirsium* as well as *Geum*-species, wanted to test the generalities which he had found in *Pisum*” [“*Aus dem 1*. *Brief Mendels an Nägeli* (*31*. *Dezember 1866*) *gewinnt man den Eindruck*, *daß er mit seinen Kreuzungen bei Hieracium*-, *Cirsium*-, *sowie Geum-Arten die bei Pisum gewonnenen Gesetzmäßigkeiten prüfen wollte*.” (p. 99)]. The wording “one gets the impression” suggests Weiling was aware of the contradiction in the letter. As far as we know, this major contradiction has never been discussed. Here we suggest that the present paragraphs four and five in Mendel’s first letter were originally not linked, but were separated by one or more lost pages. The two paragraphs are not logically connected and we propose that Mendel did not select these species to test the *Pisum* findings.

### Could a missing page explain the contradictions in Mendel’s first letter?

Because of the contradiction in Letter I, we wondered whether a part of the letter could be missing. Witte (1971), who had photocopies of all the handwritings, compared the original text with the transcript of Correns and found only a few small typographical errors. Therefore an error in the transcription can be ruled out.

**Figure 1:**
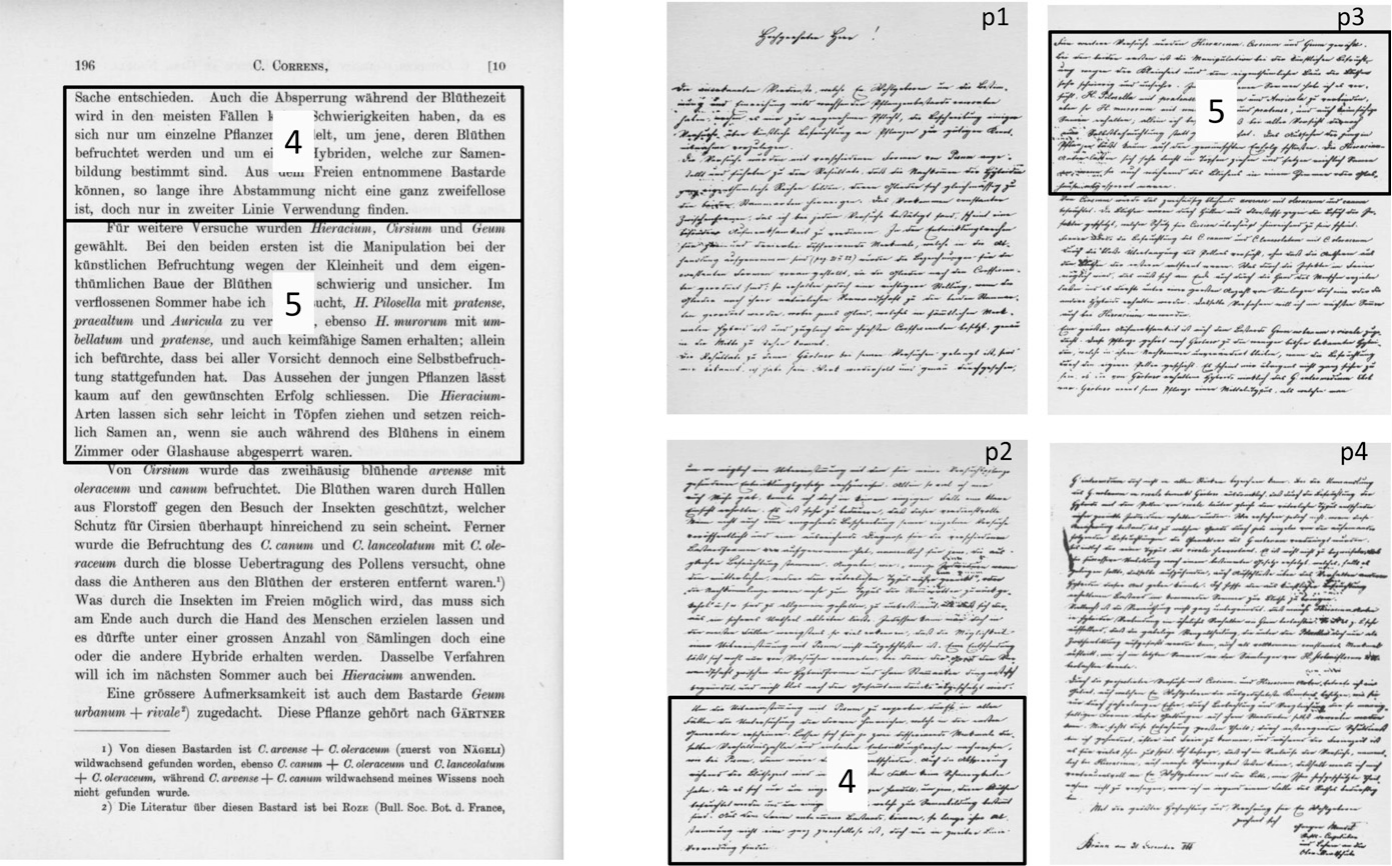
Letter I (December 31, 1866). A comparison between Correns’ publication (left) and Mendel’s original handwriting. In Correns’ publication, paragraph four and five are on the same page, but in Mendel’s original letter, paragraph four is at the end of page two and paragraph five is at the top of page three. The handwriting shows that an entire page could be missing. In Correns’ publication a missing page would not be noticed, unless the flow of the content was illogical. Courtesy of the Mendelianum Archives of the Moravian Museum.

We have examined a facsimile of Letter I (December 31, 1866) published by Jelinek (1965) because, despite our efforts, the original could not be traced. In Figure 1 it can be seen that paragraph four ends at the bottom of page two and paragraph five begins at the top of page three. Since the page break does not result in a broken sentence, a missing sheet would go unnoticed, especially in a transcript, where the relationships between paragraphs and pages are different from the original handwriting. In the facsimile, parts of the words written on page two can be seen mirror-wise on page one and vice versa; the same for pages three and four (Figure S4). This means that the sheets of paper are written on both sides and that one or more sheets could be missing (*i.e.*, two or an even number of pages). We examined copies of Mendel’s handwritten pages to see whether there were any structural clues that would enable us to discount the possibility that one or more pages is missing. From a statistical consideration of the location of page and paragraph breaks in Mendel’s letters, we concluded that paragraphs usually end in the middle of pages, so the location of a paragraph end at the bottom of a page is consistent with this being deliberate. The paragraph need not have ended there: alignment of the text using the ink marks that can be seen through the paper from one side to the other shows that there was adequate room to continue writing on this piece of paper (Section 3, File S1 and Figure S4). If paragraph five begins at the top of the page, as it does according to Correns’ transcript, then a missing page is required to end with a paragraph break. The analysis which leads us to conclude that this is not improbable is set out in Section 3, File S1.

## Mendel’s Research Interests Were Broad

### Mendel’s hypothesis about the germ cells of constant *vs.* variable hybrids

In the concluding remarks of the *Pisum* article, Mendel stressed the importance of the “essential difference” between variable and constant hybrids; between hybrids like those of pea, which produced variable offspring; and hybrids that produced constant offspring. He also mentioned that “For the history of the evolution of plants this circumstance is of special importance, since constant hybrids acquire the status of *new species*” (Mendel’s emphasis, Stern and Sherwood 1966, p. 41). By “new species” Mendel meant being true breeding and having morphological distinctness. Clearly speciation was one of the interests that Mendel had in constant hybrids.

**Figure 2:**
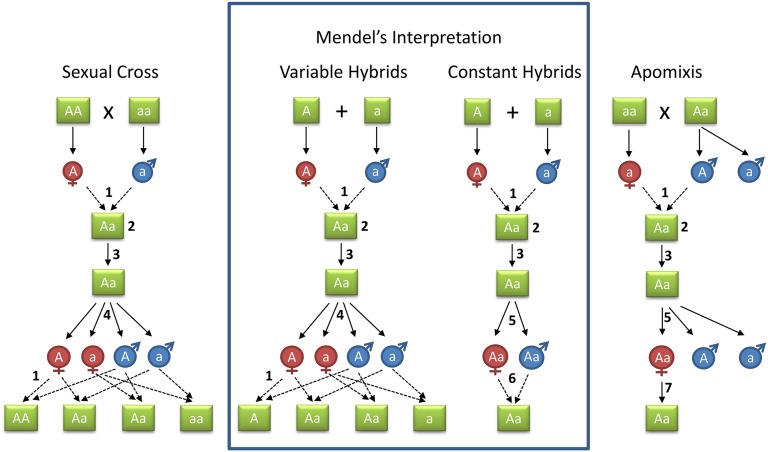
Mendel’s 1865/1866 views of inheritance in constant and variable hybrids. Mendel’s interpretation (boxed) of the behavior of determining elements is compared to our current understanding. “Sexual Cross” refers to the specific case of a cross between two homozygotes followed by self-fertilization, and should be compared to “Variable Hybrids” which is classically described in his 1866 article. Mendel’s interpretation of “Constant Hybrids” should be compared to “Apomixis.” Note that Aa has a different meaning in our current understanding from that in Mendel’s scheme; Mendel did not know about meiosis and the distinction between diploid and haploid. Numbers indicate: (1) The union of germinal cells from the female and male (egg and pollen). (2) The primordial cell (zygote): differences between antagonistic elements are mediated (in the mediating cell). (3) Vegetative period, the balance/mediation established in the primordial cell continues. (4) In variable hybrids, at the formation of the reproductive cells (gametes) the antagonistic elements are separated and represent “all constant forms which result from the combination of the characters united in fertilization.” The “arrangement between the conflicting elements is only temporary,” that is, no germinal cells carry the union of conflicting factors. (5) In constant hybrids, at the formation of the reproductive cells (gametes) the antagonistic elements are not separated. The essential difference in the development of constant hybrids is that the union of the factors is permanent. (6) In constant hybrids, the union of germinal cells of identical constitution is proposed (*i.e.*, no parthenogenesis). (7) For comparison, the genetic transmission of apomixis is shown: the unreduced egg cell develops into an embryo by parthenogenesis. Note that in the case of apomixis a breeding system is inherited, which will fix the segregating genetic background of both parents; producing many different apomictic lineages. For simplicity only diploids are shown, but apomixis is often associated with polyploidy. Because apomicts have a simplex dominant genotype (*Aaaa*) this convenience is used. The type of apomixis shown here is typical for the subgenus *Archieracium*, which Mendel also used in crosses.

Mendel was interested in the mechanisms of inheritance and the composition of reproductive cells. So far, this aspect of Mendel’s work has not received much attention. According to the report of Mendel’s second lecture on March 8, 1865 in the Brünn newspaper *Neuigkeiten*, “he spoke about cell formation, fertilization and seed production in general and in the case of hybrids in particular . . .” (Olby 1985). In his *Pisum* article, Mendel developed a hypothesis about the segregation of antagonistic elements among reproductive cells and their reassortment among progeny, based on the different types of progenies of variable and constant hybrids (Figure 2). This was >20 years before meiosis was discovered and understood by the contributions of van Beneden, Hertwig, Weismann, and others (Mayr 1982).

Mendel (1866) proposed that in variable hybrids that were derived from parents that differed, both the antagonistic elements were temporarily accommodated during the vegetative stage, and separated during the formation of the reproductive cells (egg cells and pollen). In contrast, in constant hybrids, Mendel proposed a permanent mediation. “This attempt to relate the important difference in the development of hybrids as to permanent or temporary association of differing cell elements can, of course, be of value only as a hypothesis which, for lack of well-substantiated data, still leaves some latitude.” (Stern and Sherwood 1966, p. 43). Constant hybrids, such as *Hieracium*, could provide such well-substantiated data; so, after having studied the variable *Pisum* hybrids, it was logical that Mendel would have gone on to study constant hybrids, as presaged by his comments in the *Pisum* article. Moreover, Mendel may not have been satisfied with Gärtner as an “eminent observer” as he wrote in the *Pisum* article, since in Letter I (Stern and Sherwood 1966, p. 57) to Nägeli he criticized Gärtner’s observations with respect to variable hybrids (“it is very regrettable that this worthy man did not publish a detailed description of his individual experiments”). Taken together, these considerations would have provided the impetus for Mendel to investigate constant hybrids himself.

### Mendel’s interest in *Hieracium*, *Cirsium*, and *Geum*

As he neared the completion of his *Pisum* experiments, Mendel had started looking for species for new crossing experiments. In 1864 he had made crosses between *Verbascum* and *Campanula* species and some of his artificial hybrids were shown at the June 14, 1865 meeting of the Natural Science Society (Naturforschender Verein) of Brünn. The *Verbascum* hybrids, however, were completely sterile (Letter III, Stern and Sherwood 1966, p. 77). The timing shows that Mendel’s interest in variable hybrids continued while he was also studying constant hybrids.

Why did Mendel select *Hieracium*, *Geum*, and *Cirsium*? Mendel mentioned in Letter I that the artificial hybrid Gärtner had made between *Geum urbanum* and *Geum rivale* was one of the few hybrids known so far that produced constant progeny plants. Both parental species showed discrete alternative states of traits, which had been a methodological requirement for Mendel’s study of variable hybrids. Moreover, the taxon *G. intermedium* was found in nature, which could be the constant hybrid between *G. urbanum* and *G. rivale*. The last page of Mendel’s personal copy of Gärtner’s (1849) *Versuche und Beobachtungen über die Bastarderzeugung im Pflanzenreich* (Experiments and Observations on Hybridization in the Plant Kingdom) contains many notes on *Geum*, and two interesting designations of multigene genotypes of *G. intermedium*: *ABcDEe* and *ABcdEe* (Olby 1985). In these, the heterozygote *Ee* would be constant and would not segregate.

Mendel was an active member of the Natural Science Society where he gave the two 1865 lectures about his *Pisum* experiments. In 1869, he was elected as vice president of the society and in June of that year he gave a lecture about his *Hieracium* hybridization experiments. Both *Hieracium* and *Cirsium* were genera in which intermediate and transitional forms between species were common (Nägeli 1866). Nägeli speculated that these might be constant hybrids or products of transmutation. Natural hybrids of *Hieracium* and *Cirsium* had already been discussed at several meetings of the society (see Section 4, File S1; Weiling 1969; Orel 1996). In general, the society was more interested in interspecific hybridization (“*Bastarde*”), than in intraspecific hybridization (“*Hybriden*”). Although Mendel saw only a graduated difference between varieties and species, he used “*Hybriden*” in the title of his *Pisum* article and “*Bastarde*” in the title of his *Hieracium* article; showing that he was well aware of the difference. His interest in species *vs.* varieties may have been influenced by the publication of Darwin’s (1859) *Origin of Species* [Mendel had a copy of the second edition of the German translation of the *Origin of Species* (1863), see Fairbanks and Rytting 2001]. Mendel’s selection of *Hieracium*, *Geum*, and *Cirsium* for study is therefore something to be expected in the intellectual atmosphere of Brünn at that time.

## Hieracium

### Two phases of Mendel’s *Hieracium* research

Mendel’s letters to Nägeli give a unique insight into his character, showing the evolution of his views, his openness and honesty, and his admission that some of his earlier expectations were incorrect. In some places the letters are witty and self-deprecating. Also striking, and contrary to what is often claimed, the correspondence between Mendel and Nägeli is friendly: Nägeli was not arrogant or controlling toward Mendel (Schwartz, 2008, and see salutations and signings Table S1). Although Mendel wrote about experiments with other species, in these letters the *Hieracium* experiments were by far the most important. *Geum* and *Cirsium* did not produce constant hybrids and soon Mendel concentrated on *Hieracium*. Mendel’s letters and his provisional *Hieracium* communication makes it possible to reconstruct his *Hieracium* crossing experiments (see Table S2 for a timeline, and Table S3 in relation to Mendel’s interspecific crosses). A large part of the correspondence is about the identification of *Hieracium* species and the exchanges of plant material, which, although they were important at the time, obscure the purpose of the investigation.

Based on the content of the correspondence, two research phases can be distinguished (see Section 5, File S1); in the first phase Mendel, with great effort, managed to produce some hybrids which indeed propagated constantly. The preliminary communication on *Hieracium* hybrids of June 9, 1869 can be seen to conclude this phase. In the second phase, Mendel tried to find a solution to the fact that, contrary to his expectation, he found multiple types of constant hybrid. Nogler (2006) gives a good biological description and analysis of Mendel’s *Hieracium* experiments, although it is chronologically incorrect. He wrote that Mendel was first surprised by the many different F_1_ hybrids and then by the fact that these hybrids were true breeding. This chronology reinforced the image of a frustrated Mendel. In reality, Mendel initially obtained very few hybrids. It must have been an exciting vindication that the first hybrid was true breeding, fulfilling his *Sehnsucht*. Only later, to his surprise, he found that there were many different but constant F_1_ hybrids. In total, Mendel obtained hybrids in 21 interspecific combinations. Table S3 lists the most important interspecific hybrids and the variability of their offspring.

**Figure 3:**
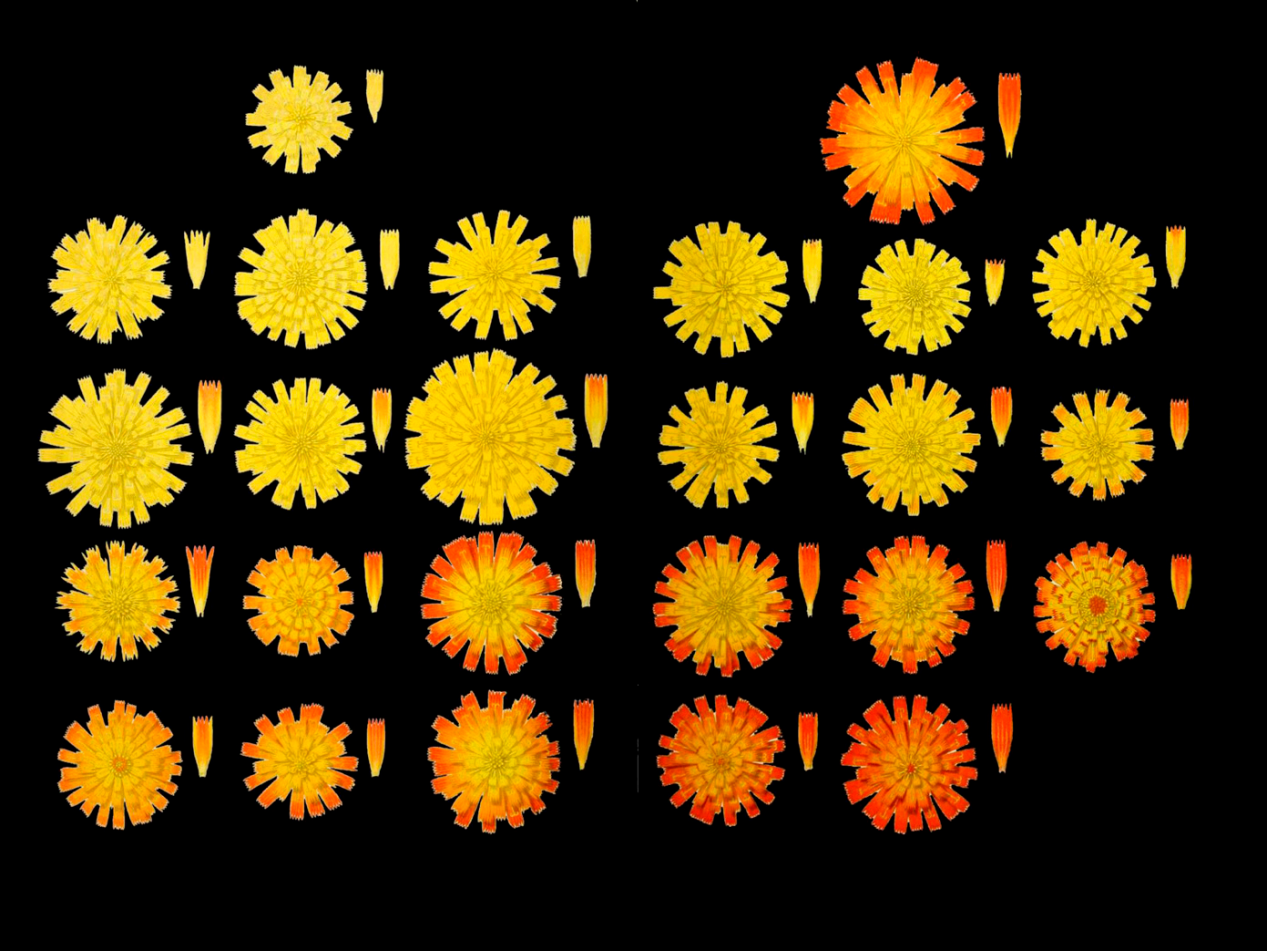
Variation in inflorescence color and size in *Hieracium* hybrids. Ostenfeld (1910) illustrated 23 *H. auricula* × *aurantiacum* hybrids that he obtained. Mendel obtained 84 flowering hybrids from the same cross. The parental species are shown at the top; *H. auricula* left, with a yellow small inflorescence; and *H. aurantiacum* right, with a larger orange inflorescence. Next to the inflorescence a single floret is shown. The original image is from the Biodiversity Heritage Library. Digitized by the Mertz Library, New York Botanical Garden (http://www.biodiversitylibrary.org).

**Figure 4:**
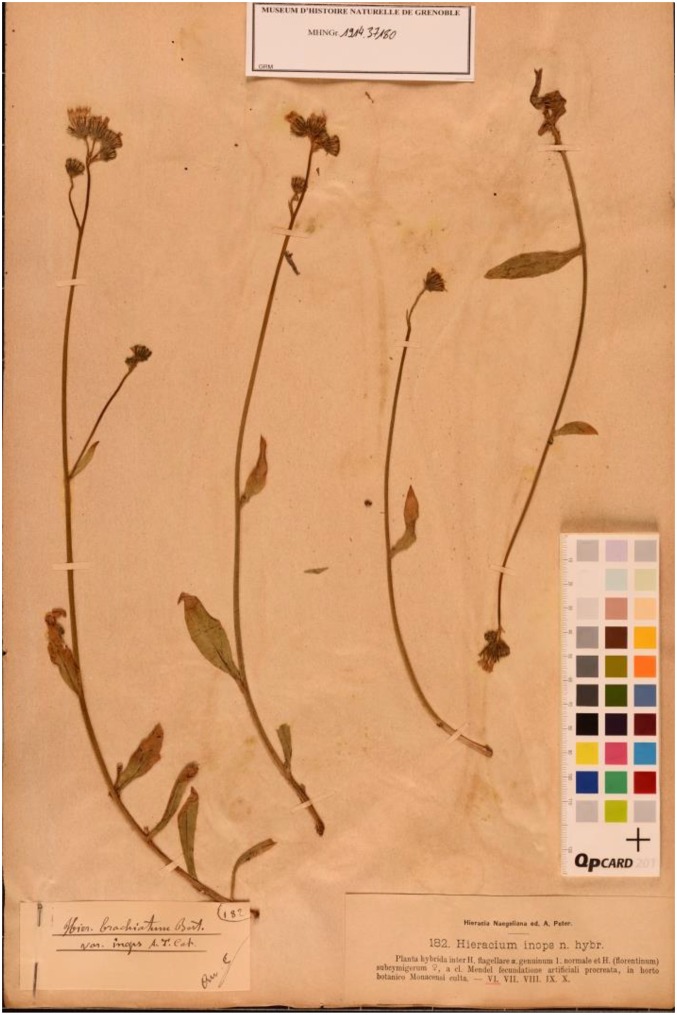
Mendel’s first constant *Hieracium* hybrid (*H. praealtum* × *H. flagellare*). Mendel observed no variation in three generations of this artificial hybrid. From herbarium “*Hieracia Naegeliana*” (Peter 1885). Courtesy of the Museum of Grenoble (*H. inops* n. hybr., GRM. Arv.-Touv. MHNGr. 191437180).

**Figure 5:**
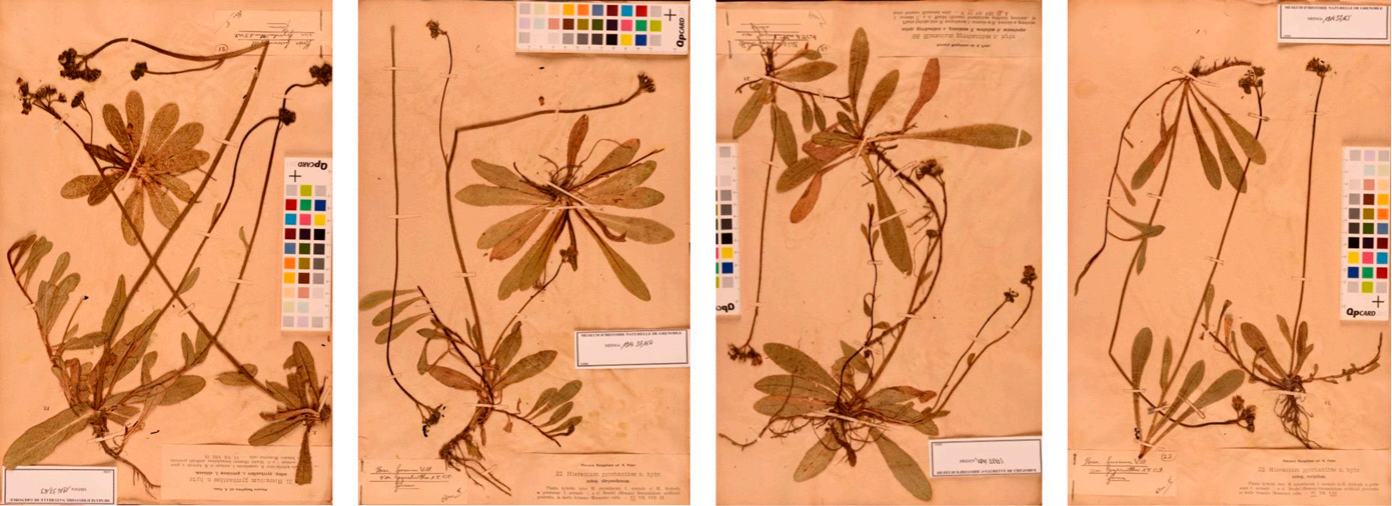
Four of Mendel’s *H. auricula* × *aurantiacum* hybrids from the herbarium *Hieracia Naegeliana* (Peter 1885). All hybrids were fully fertile. In 1869, 1870, and 1873 Mendel sent material to Nägeli in Munich where they were cultivated in the common garden. Eight of these are described in Peter (1884). Courtesy of Museum de Grenoble (*H. pyrrhanthes* n. hybr. GRM. Arv.-Touv. MHNGr. 191437163, 191437164, 191437165, and 191437173).

Mendel’s most successful cross was that between *H. auricula* × *aurantiacum* from which he obtained 84 fertile hybrids (40 years later Ostenfeld repeated this cross, Figure 3). Remarkably, some of Mendel’s hybrids still exist as dried specimens in the Herbarium of the Museum of Grenoble (Mendel’s first constant hybrid, Figure 4; several *H. auricula* × *aurantiacum* hybrids, Figure 5). The hybrids that Mendel sent to Nägeli were grown in the experimental garden of the University of Munich. Nägeli’s student and later colleague, Albert Peter, edited a collection of exsiccatae “*Hieracia Naegeliana*” (1885), consisting of 300 herbarium sheets of *Hieracium* subgenus *Pilosella* plants, which included 16 of Mendel’s hybrids and 12 parental forms. Weiling (1969) located the “*Hieracia Naegeliana*” in 23 other herbaria in 11 countries throughout Europe, although these are often incomplete.

In the first phase of Mendel’s *Hieracium* experiments, he demonstrated the constancy of the hybrid in subsequent generations. He could have hoped to use this, for example, to study dominance relationships among determinants for the differentiating characters. However, the observation of more than one type of constant hybrid was unexpected because the parents were also true breeding and only one F_1_ hybrid type was anticipated. The second phase of the *Hieracium* experiments was therefore to determine what caused the multiplicity of F_1_ types. Mendel knew from his *Pisum* methodology that he should collect very many F_1_ hybrids to “determine the number of different forms in which the hybrid progeny appear . . . and ascertain their numerical interrelationships” (Stern and Sherwood 1966, p. 2). He was well aware of the amount of work this would require and in trying to improve the efficiency of the microscopic *Hieracium* crosses he nearly ruined his eyesight permanently. In his final letter to Nägeli, reflecting his realization that he did not have sufficient time to complete the necessary experiments, he wrote: “I am really unhappy about having to neglect my plants and my bees so completely. Since I have a little spare time at present, and since I do not know whether I shall have any next spring, I am sending you today some material from my last experiments in 1870 and 1871.” (Letter X, Stern and Sherwood 1966, p. 97). All he could do was pass on his experimental material to someone who may have the opportunity to continue the work. If he was frustrated, it was not because his experiments had failed, but because they showed what needed to be done next and his duties as abbot prevented him from continuing this work.

## Concluding Remarks

In this article we have argued that Mendel’s *Hieracium* experiments, and the reasons underlying them, have been misunderstood for more than a century. We propose that this misunderstanding rests on the obscurity of the originals of his written letters and that a missing page (or pages) in his first letter to Nägeli would explain the common misreading of that letter. There is no proof that a page is missing; this could become a certainty only if it were found, which seems highly unlikely. Notwithstanding, the traditional view of Mendel’s *Hieracium* experiments is not the only one possible. The interpretation we set out here is consistent with the whole of Mendel’s known writings and does not involve the contradiction necessary for the traditional view. We therefore consider our interpretation the more likely. A missing page is not a necessary requirement for our interpretation, but its suggested location would help to explain the prolonged misinterpretation.

Although Mendel’s letters to Nägeli mainly concern the *Hieracium* crosses, as would be expected because of their collaboration, the letters also contain important information about his variable hybrids and this has been neglected, perhaps because of the negative view of his *Hieracium* work. In July 1870 (Letter VIII), Mendel wrote: “Of the experiments of previous years, those dealing with *Matthiola annua* and *glabra*, *Zea*, and *Mirabilis* were concluded last year. Their hybrids behave exactly like those of *Pisum*. Darwin’s statements concerning hybrids of the genera mentioned in *The Variation of Animals and Plants Under Domestication*, based on reports of others, need to be corrected in many respects.” (Stern and Sherwood 1966, p. 93). This clearly shows that Mendel had found additional support for his understanding of inheritance in variable hybrids. In the same letter and in the next (Letter IX, September 27, 1870), Mendel also described repeated experiments to test whether a single pollen grain is sufficient to fertilize a single egg cell and an experiment with two pollen grains, each from a different flower color genotype, to investigate if an egg cell could be fertilized by two pollen grains simultaneously. These experiments are a rigorous test of the basic principles of his theory of inheritance in *Pisum*. Contrary to the historians’ view, there can be no doubt that Mendel was above all a geneticist.

“My time is yet to come” are the famous prophetic words attributed to Mendel by his friend Gustav von Niessl. It is not widely known that Mendel said these words in the garden among his *Hieracium* and *Cirsium* plants. (“*aber ich hörte im Garten*, *an den Beeten seiner Hieracien und Cirsien von ihm die prophetischen Worte:* ‘*Meine Zeit wird noch kommen*,’ ” Von Niessl 1905, p. 8). A more appropriate location is hard to imagine. Mendel’s interest in hybrids (both inter- and intraspecific) was broadly based and encompassed the mechanism of their formation, inheritance in general, as well as the consequences of hybridization for evolution. He clearly recognized two contrasting types of hybrid (constant and variable) and he chose to study both. In one of his last letters to Nägeli, he commented: “Evidently we are here dealing only with individual phenomena, which are the manifestation of a higher, more fundamental, law” (Stern and Sherwood 1966, p. 90). With hindsight we see this to be entirely correct. Mendel’s observations in *Hieracium* demonstrated the pollen transmission of apomixis that can now be understood in terms of the Mendelian genetics of the process of inheritance itself.

## 

## Footnotes

1 By “constant hybrids,” Mendel means true-breeding *Aa* hybrids. In modern genetic terms these are heterozygotes that remain heterozygotes in subsequent generations. This must be clearly distinguished from true-breeding new trait combinations in variable hybrids (*e.g*., *AAbb*, *aaBB*).

2 For Mendel, the “first generation” referred to the first generation derived from the hybrid—today this would be called the F_2_.

3 The wording “further experiments” (“*weitere Versuche*”) is somewhat awkward or ambiguous in this context. Since Mendel gave a detailed protocol as to how the *Pisum* findings could be tested in the previous paragraph, his having written “*Hieracium*, *Cirsium*, and *Geum* I have selected for further experiments” rather than “. . . such experiments” might suggest he was referring to a different kind of experiment.

4 “*bisher*” (meaning “so far”) was not translated by Piternick and Piternick (1950). However, it indicates that Mendel expected that more constant hybrids would be found, which is logical if he already suspected *Hieracium* hybrids to be constant.

5 Piternick and Piternick (1950) use “transitional,” but we think “intermediate” is a better translation of “*Zwischenbildung*.”

6 Nägeli (1845) mentioned a forked stem as a characteristic of *Pilosella* hybrids.
